# Supplementary material for: Modeling glioblastoma heterogeneity as a dynamic network of cell states
Source: Mol Syst Biol. 2021 Sep 16;17(9):e10105. doi: 10.15252/msb.202010105 (PMC8444284; doi:10.15252/msb.202010105)
Supplement: Supplementary file 5 — Source Data for Figure 3 [file MSB-17-e10105-s001.zip › Figure3A_sourcedata/GSEA_3065/nosigdb_state1.GseaPreranked.1622718409535/NB_KI_CLUSTER3.html]

Details for gene set NB\_KI\_CLUSTER3[GSEA]

|  || Dataset | state1 |
| Phenotype | NoPhenotypeAvailable |
| Upregulated in class | na\_pos |
| GeneSet | NB\_KI\_CLUSTER3 |
| Enrichment Score (ES) | 0.5663479 |
| Normalized Enrichment Score (NES) | 2.1499546 |
| Nominal p-value | 0.0 |
| FDR q-value | 0.0 |
| FWER p-Value | 0.0 |
Table: GSEA Results Summary

  

Fig 1: Enrichment plot: NB\_KI\_CLUSTER3      
 Profile of the Running ES Score & Positions of GeneSet Members on the Rank Ordered List

  

| PROBE | GENE SYMBOL | GENE\_TITLE | RANK IN GENE LIST | RANK METRIC SCORE | RUNNING ES | CORE ENRICHMENT || 1 | ODC1 |  |  | 7 | 0.744 | 0.0350 | Yes |
| 2 | HMGA1 |  |  | 8 | 0.736 | 0.0702 | Yes |
| 3 | TXN |  |  | 13 | 0.686 | 0.1027 | Yes |
| 4 | PKM |  |  | 14 | 0.676 | 0.1351 | Yes |
| 5 | TPI1 |  |  | 52 | 0.473 | 0.1540 | Yes |
| 6 | PPP1R14B |  |  | 69 | 0.418 | 0.1724 | Yes |
| 7 | ARPC1A |  |  | 87 | 0.385 | 0.1891 | Yes |
| 8 | ACTG1 |  |  | 94 | 0.380 | 0.2067 | Yes |
| 9 | ENO1 |  |  | 99 | 0.373 | 0.2241 | Yes |
| 10 | AKR1B1 |  |  | 100 | 0.372 | 0.2420 | Yes |
| 11 | CFL1 |  |  | 138 | 0.329 | 0.2539 | Yes |
| 12 | YBX1 |  |  | 146 | 0.326 | 0.2688 | Yes |
| 13 | SLC25A4 |  |  | 157 | 0.318 | 0.2830 | Yes |
| 14 | HRAS |  |  | 159 | 0.317 | 0.2981 | Yes |
| 15 | UBE2M |  |  | 170 | 0.310 | 0.3120 | Yes |
| 16 | ARHGDIA |  |  | 185 | 0.303 | 0.3251 | Yes |
| 17 | PGAM1 |  |  | 190 | 0.302 | 0.3391 | Yes |
| 18 | CYCS |  |  | 203 | 0.294 | 0.3520 | Yes |
| 19 | MRPL33 |  |  | 211 | 0.291 | 0.3652 | Yes |
| 20 | EIF4EBP1 |  |  | 216 | 0.290 | 0.3787 | Yes |
| 21 | MRPL17 |  |  | 226 | 0.285 | 0.3914 | Yes |
| 22 | EIF5A |  |  | 308 | 0.258 | 0.3955 | Yes |
| 23 | ACOT7 |  |  | 309 | 0.258 | 0.4078 | Yes |
| 24 | COPRS |  |  | 369 | 0.235 | 0.4130 | Yes |
| 25 | NDUFAF8 |  |  | 392 | 0.228 | 0.4217 | Yes |
| 26 | HSPD1 |  |  | 397 | 0.227 | 0.4321 | Yes |
| 27 | COX5B |  |  | 400 | 0.226 | 0.4427 | Yes |
| 28 | ARPC5L |  |  | 409 | 0.224 | 0.4526 | Yes |
| 29 | TUBG1 |  |  | 439 | 0.219 | 0.4601 | Yes |
| 30 | CACYBP |  |  | 458 | 0.214 | 0.4685 | Yes |
| 31 | RAC1 |  |  | 472 | 0.211 | 0.4773 | Yes |
| 32 | ANAPC11 |  |  | 490 | 0.208 | 0.4855 | Yes |
| 33 | EIF3I |  |  | 505 | 0.205 | 0.4939 | Yes |
| 34 | INPP5F |  |  | 518 | 0.201 | 0.5023 | Yes |
| 35 | FKBP4 |  |  | 547 | 0.196 | 0.5088 | Yes |
| 36 | TAF9 |  |  | 596 | 0.186 | 0.5128 | Yes |
| 37 | SET |  |  | 634 | 0.181 | 0.5177 | Yes |
| 38 | HMGN1 |  |  | 737 | 0.167 | 0.5152 | Yes |
| 39 | CISD1 |  |  | 757 | 0.165 | 0.5211 | Yes |
| 40 | PSMC4 |  |  | 803 | 0.157 | 0.5240 | Yes |
| 41 | PRMT1 |  |  | 884 | 0.146 | 0.5228 | Yes |
| 42 | CAMTA1 |  |  | 905 | 0.144 | 0.5276 | Yes |
| 43 | CASP3 |  |  | 923 | 0.142 | 0.5327 | Yes |
| 44 | SH3GLB2 |  |  | 978 | 0.137 | 0.5337 | Yes |
| 45 | RPL37A |  |  | 979 | 0.136 | 0.5402 | Yes |
| 46 | RFK |  |  | 981 | 0.135 | 0.5466 | Yes |
| 47 | FAM104A |  |  | 1006 | 0.133 | 0.5505 | Yes |
| 48 | GARS |  |  | 1021 | 0.132 | 0.5554 | Yes |
| 49 | MAGOHB |  |  | 1047 | 0.128 | 0.5590 | Yes |
| 50 | SNRPC |  |  | 1069 | 0.126 | 0.5628 | Yes |
| 51 | COA3 |  |  | 1119 | 0.121 | 0.5636 | Yes |
| 52 | NT5DC2 |  |  | 1155 | 0.118 | 0.5656 | Yes |
| 53 | CCDC25 |  |  | 1239 | 0.109 | 0.5623 | Yes |
| 54 | SSX2IP |  |  | 1331 | 0.100 | 0.5577 | Yes |
| 55 | GOT1 |  |  | 1344 | 0.100 | 0.5613 | Yes |
| 56 | LCMT1 |  |  | 1438 | 0.093 | 0.5561 | Yes |
| 57 | SRP14 |  |  | 1445 | 0.093 | 0.5600 | Yes |
| 58 | SLC25A39 |  |  | 1459 | 0.092 | 0.5630 | Yes |
| 59 | FABP5 |  |  | 1470 | 0.091 | 0.5663 | Yes |
| 60 | NELFE |  |  | 1518 | 0.088 | 0.5657 | No |
| 61 | UBB |  |  | 1620 | 0.081 | 0.5592 | No |
| 62 | HDGFL2 |  |  | 1664 | 0.078 | 0.5585 | No |
| 63 | GPN1 |  |  | 1718 | 0.074 | 0.5566 | No |
| 64 | MANBAL |  |  | 1741 | 0.073 | 0.5578 | No |
| 65 | SUCLG1 |  |  | 1795 | 0.070 | 0.5557 | No |
| 66 | PSMB4 |  |  | 1820 | 0.068 | 0.5565 | No |
| 67 | DBNL |  |  | 1831 | 0.068 | 0.5588 | No |
| 68 | EXOSC8 |  |  | 1851 | 0.067 | 0.5600 | No |
| 69 | SEPHS2 |  |  | 1863 | 0.067 | 0.5621 | No |
| 70 | DDX1 |  |  | 1984 | 0.060 | 0.5527 | No |
| 71 | TRAP1 |  |  | 2011 | 0.059 | 0.5528 | No |
| 72 | JPT2 |  |  | 2015 | 0.059 | 0.5553 | No |
| 73 | UTP18 |  |  | 2020 | 0.059 | 0.5577 | No |
| 74 | BAZ1B |  |  | 2108 | 0.055 | 0.5514 | No |
| 75 | WDR18 |  |  | 2169 | 0.052 | 0.5477 | No |
| 76 | ACYP1 |  |  | 2324 | 0.046 | 0.5341 | No |
| 77 | GPS1 |  |  | 2333 | 0.046 | 0.5355 | No |
| 78 | MRPL10 |  |  | 2359 | 0.045 | 0.5350 | No |
| 79 | SLC25A17 |  |  | 2373 | 0.044 | 0.5358 | No |
| 80 | NDUFAF6 |  |  | 2442 | 0.042 | 0.5308 | No |
| 81 | GPSM2 |  |  | 2477 | 0.040 | 0.5293 | No |
| 82 | EIF4E |  |  | 2517 | 0.039 | 0.5271 | No |
| 83 | HSP90AA1 |  |  | 2562 | 0.038 | 0.5244 | No |
| 84 | PRPSAP2 |  |  | 2604 | 0.036 | 0.5219 | No |
| 85 | RRP7A |  |  | 2615 | 0.036 | 0.5226 | No |
| 86 | MRPL44 |  |  | 2624 | 0.035 | 0.5235 | No |
| 87 | UNG |  |  | 2727 | 0.032 | 0.5145 | No |
| 88 | CARHSP1 |  |  | 2827 | 0.030 | 0.5057 | No |
| 89 | DLD |  |  | 2976 | 0.025 | 0.4917 | No |
| 90 | FXR2 |  |  | 3035 | 0.024 | 0.4869 | No |
| 91 | GIHCG |  |  | 3040 | 0.024 | 0.4877 | No |
| 92 | VKORC1L1 |  |  | 3092 | 0.023 | 0.4835 | No |
| 93 | COPS7B |  |  | 3242 | 0.020 | 0.4691 | No |
| 94 | METAP2 |  |  | 3395 | 0.016 | 0.4542 | No |
| 95 | DNAJC2 |  |  | 3593 | 0.012 | 0.4345 | No |
| 96 | CBWD2 |  |  | 3628 | 0.011 | 0.4316 | No |
| 97 | DUS1L |  |  | 3705 | 0.010 | 0.4242 | No |
| 98 | PRR3 |  |  | 3718 | 0.010 | 0.4234 | No |
| 99 | PPP1R35 |  |  | 3774 | 0.009 | 0.4182 | No |
| 100 | NDUFA4 |  |  | 3842 | 0.007 | 0.4116 | No |
| 101 | CCDC167 |  |  | 3921 | 0.006 | 0.4039 | No |
| 102 | NEK1 |  |  | 4071 | 0.003 | 0.3887 | No |
| 103 | HIRIP3 |  |  | 4163 | 0.001 | 0.3794 | No |
| 104 | SINHCAF |  |  | 4175 | 0.001 | 0.3783 | No |
| 105 | HSPA8 |  |  | 4292 | -0.001 | 0.3664 | No |
| 106 | FLAD1 |  |  | 4464 | -0.004 | 0.3490 | No |
| 107 | HPF1 |  |  | 4607 | -0.006 | 0.3347 | No |
| 108 | SPOP |  |  | 4653 | -0.007 | 0.3304 | No |
| 109 | NUFIP1 |  |  | 4665 | -0.007 | 0.3296 | No |
| 110 | VPS72 |  |  | 4685 | -0.007 | 0.3280 | No |
| 111 | LDHB |  |  | 4712 | -0.008 | 0.3257 | No |
| 112 | NLN |  |  | 4753 | -0.008 | 0.3220 | No |
| 113 | MFAP1 |  |  | 4974 | -0.012 | 0.2999 | No |
| 114 | SCCPDH |  |  | 5110 | -0.014 | 0.2867 | No |
| 115 | HSF2 |  |  | 5188 | -0.015 | 0.2795 | No |
| 116 | ASNS |  |  | 5370 | -0.018 | 0.2617 | No |
| 117 | SYNC |  |  | 5372 | -0.018 | 0.2625 | No |
| 118 | CTDSPL2 |  |  | 5477 | -0.020 | 0.2527 | No |
| 119 | METTL2A |  |  | 5707 | -0.024 | 0.2303 | No |
| 120 | TOPBP1 |  |  | 5733 | -0.024 | 0.2289 | No |
| 121 | WDR54 |  |  | 5844 | -0.026 | 0.2188 | No |
| 122 | PARP2 |  |  | 5986 | -0.029 | 0.2057 | No |
| 123 | ACTR6 |  |  | 6153 | -0.032 | 0.1902 | No |
| 124 | CTCF |  |  | 6233 | -0.034 | 0.1836 | No |
| 125 | ENOPH1 |  |  | 6568 | -0.040 | 0.1512 | No |
| 126 | ADAT1 |  |  | 6587 | -0.040 | 0.1513 | No |
| 127 | MSH6 |  |  | 6591 | -0.040 | 0.1529 | No |
| 128 | ZNF620 |  |  | 6617 | -0.041 | 0.1523 | No |
| 129 | POLE |  |  | 6848 | -0.046 | 0.1308 | No |
| 130 | ATP6V0B |  |  | 6884 | -0.047 | 0.1295 | No |
| 131 | CENPJ |  |  | 7066 | -0.052 | 0.1133 | No |
| 132 | DCAKD |  |  | 7092 | -0.053 | 0.1133 | No |
| 133 | COMMD2 |  |  | 7125 | -0.054 | 0.1126 | No |
| 134 | FIGNL1 |  |  | 7176 | -0.055 | 0.1100 | No |
| 135 | IFT22 |  |  | 7211 | -0.056 | 0.1092 | No |
| 136 | CCP110 |  |  | 7307 | -0.058 | 0.1022 | No |
| 137 | SSBP2 |  |  | 7335 | -0.059 | 0.1023 | No |
| 138 | SNX4 |  |  | 7350 | -0.059 | 0.1037 | No |
| 139 | KLHL23 |  |  | 7508 | -0.064 | 0.0906 | No |
| 140 | CPSF3 |  |  | 7593 | -0.066 | 0.0851 | No |
| 141 | CEP78 |  |  | 7627 | -0.067 | 0.0849 | No |
| 142 | FANCL |  |  | 7647 | -0.068 | 0.0862 | No |
| 143 | CDKN2C |  |  | 7660 | -0.068 | 0.0882 | No |
| 144 | NSUN5 |  |  | 7670 | -0.069 | 0.0906 | No |
| 145 | ZGRF1 |  |  | 7737 | -0.071 | 0.0872 | No |
| 146 | KLHL42 |  |  | 7800 | -0.073 | 0.0844 | No |
| 147 | SEC61A2 |  |  | 7931 | -0.079 | 0.0747 | No |
| 148 | SFXN1 |  |  | 7952 | -0.079 | 0.0765 | No |
| 149 | RNF219 |  |  | 8077 | -0.084 | 0.0678 | No |
| 150 | FSD1L |  |  | 8306 | -0.095 | 0.0488 | No |
| 151 | KNOP1 |  |  | 8340 | -0.096 | 0.0501 | No |
| 152 | VPS45 |  |  | 8499 | -0.105 | 0.0388 | No |
| 153 | MMS22L |  |  | 8547 | -0.108 | 0.0392 | No |
| 154 | SLC25A13 |  |  | 8672 | -0.115 | 0.0319 | No |
| 155 | FADS1 |  |  | 9044 | -0.147 | 0.0008 | No |
| 156 | ZNF519 |  |  | 9134 | -0.156 | -0.0009 | No |
| 157 | ZNF738 |  |  | 9407 | -0.201 | -0.0192 | No |
| 158 | HLTF |  |  | 9410 | -0.204 | -0.0097 | No |
| 159 | SGCB |  |  | 9520 | -0.231 | -0.0098 | No |
| 160 | TRIM24 |  |  | 9561 | -0.245 | -0.0022 | No |
| 161 | HACD3 |  |  | 9697 | -0.311 | -0.0012 | No |
| 162 | RTN3 |  |  | 9792 | -0.413 | 0.0090 | No |
Table: GSEA details [plain text format]

  

Fig 2: NB\_KI\_CLUSTER3: Random ES distribution      
 Gene set null distribution of ES for **NB\_KI\_CLUSTER3**
